# Co-Loading of Inorganic Nanoparticles and Natural Oil in the Electrospun Janus Nanofibers for a Synergetic Antibacterial Effect

**DOI:** 10.3390/pharmaceutics14061208

**Published:** 2022-06-06

**Authors:** Menglong Wang, Deng-Guang Yu, Gareth R. Williams, Sim Wan Annie Bligh

**Affiliations:** 1 School of Health Sciences, Caritas Institute of Higher Education, Hong Kong 999077, China; 191370148@st.usst.edu.cn; 2 School of Materials and Chemistry, University of Shanghai for Science and Technology, Shanghai 200093, China; 3 UCL School of Pharmacy, University College London, London WC1E 6BT, UK; g.williams@ucl.ac.uk

**Keywords:** side-by-side electrospinning, electrospun microfibers, essential oil, Ag nanoparticles, antibacterial

## Abstract

Side-by-side electrospinning is a powerful but challenging technology that can be used to prepare Janus nanofibers for various applications. In this work, cellulose acetate (CA) and polycaprolactone (PCL) were used as polymer carriers for silver nanoparticles (Ag NPs) and lavender oil (LO), respectively, processing these into two-compartment Janus fibers. A bespoke spinneret was used to facilitate the process and prevent the separation of the working fluids. The process of side-by-side electrospinning was recorded with a digital camera, and the morphology and internal structure of the products were characterized by electron microscopy. Clear two-compartment fibers are seen. X-ray diffraction patterns demonstrate silver nanoparticles have been successfully loaded on the CA side, and infrared spectroscopy indicates LO is dispersed on the PCL side. Wetting ability and antibacterial properties of the fibers suggested that PCL-LO//CA-Ag NPs formulation had strong antibacterial activity, performing better than fibers containing only one active component. The PCL-LO//CA-Ag NPs had a 20.08 ± 0.63 mm inhibition zone for E. coli and 19.75 ± 0.96 mm for S. aureus. All the fibers had water contact angels all around 120°, and hence, have suitable hydrophobicity to prevent water ingress into a wound site. Overall, the materials prepared in this work have considerable promise for wound healing applications.

## 1. Introduction

Dressings play an important role in the treatment of wounds, acting both to prevent infection and accelerate healing. In recent decades, a range of novel wound dressings has been developed based on technologies such as sponges [1,2], hydrogels [3,4], fibers [5,6,7,8], and particles [9,10], among others. Unlike traditional wound dressings, various functions have been added to these new formulations, such as anti-inflammatory, anti-bacterial, and/or anti-adhesion properties, the ability to absorb tissue exudate, and the inclusion of active ingredients for the promotion of wound healing [11,12]. Among these properties, antibacterial performance is important for wound dressing. As bacteria can lead to a series of symptoms in the wound and prolong the healing period, it should be eliminated first and kept away from the wound. Thus, various small-molecule substances, which have antibacterial performance, including organic and inorganic, are applied in wound dressings for that purpose. Drug-loaded fibers have been applied as dressings in a number of studies [13,14,15]. Such fibers can offer controlled drug release and have huge potential as wound dressing [16,17,18]. Of all of the various fiber preparation methods, the “one-step” electrospinning technology has been frequently favored by researchers [19,20].

Electrospinning has emerged as an important technology in preparing fiber materials for a wide variety of scientific applications [21,22,23,24]. In the biomedical field, the potential applications include drug delivery systems [25,26,27,28], tissue engineering [29,30,31,32], or biosensors [33]. For fabricating advanced and multi-functional fibers, multifluid electrospinning processes have been developed in various forms [34,35,36,37,38]. To date, side-by-side electrospinning (or Janus) electrospinning has proven to be a challenging technology. However, it has many benefits since it can produce two-sided Janus fibers, with the two sides having very different properties and retaining their intrinsic properties [39]. In a traditional side-by-side electrospinning process, two parallel metallic capillaries are assembled side-by-side to form a spinneret [40]. However, electrostatic repulsion between the working fluids when they exit the spinneret can result in their separation. There are some strategies to solve this problem. One is to increase the contact area between the working fluids before the Taylor cone is formed [41,42]. The other is to use electrostatic force to bind the working fluids together [43]. Herein, a home-made Janus spinneret, comprising two 21G needles wrapped with a metal tube, was used to carry out side-by-side electrospinning. The two smaller needles are kept parallel, and the outer needle protrudes over the 21G needles. This spinneret ensures that the working fluids are in full contact before the Taylor cone is formed and helps to increase the contact area between the fluids.

The biodegradable and resorbable polymers, such as poly-ε-caprolactone (PCL) and poly(α-L-glutamic acid), have been widely used in bio-applications such as tissue engineering [44,45], drug delivery [46,47], wound dressings [48,49], scaffolds [50] and surgical sutures [51,52]. However, PCL is hydrophobic. This makes it a useful material for anti-adhesive applications but is poor at absorbing the exudate from a wound. For an effective dressing, therefore, a hygroscopic water-absorbent material is required. Cellulose acetate (CA) is one suitable material for this application: it has good water absorption and liquid transport ability, is biocompatible and, like PCL, has been used in wound dressings [53,54], tissue engineering [55], and drug delivery [56,57]. The literature reports that a mixture of PCL and CA can be processed into nanofibers through uniaxial electrospinning, and the resultant mat is impregnated with propolis. However, after treatment, the PCL/CA mats almost lost any hydrophobic properties [58]. In this work, we sought to use side-by-side electrospinning to overcome this issue.

Lavender oil (LO) is an essential oil that can be used directly on the skin and is also commonly used in wound dressings owing to its antibacterial properties and promotion of wound healing [59]. LO prevents adhesion of a dressing to the wound and thus avoids secondary injuries being caused during the changing of a dressing. Essential oils in wound dressings have been reported by many researchers. For example, Ardekani et al. used poly (vinyl alcohol) loaded with zataria multiflora essential oil to this end [60]. In another work, Unalan et al. incorporated essential clove oil into PCL/gelatin nanofibers for antibacterial wound dressings [61], while Sara et al. loaded essential oils on electrospun PCL nanofibers to generate anti-inflammatory patches [62]. As the combined antibacterial activity often performed better than one and took advantage of side-by-side electrospinning, antibacterial nanoparticles were incorporated into fibers as well.

Another material often incorporated into wound dressings is silver nanoparticles (Ag NPs), which also have antibacterial properties [63,64]. For instance, Gao et al. developed a stem cell-seeded bilayer Ag-poly(lactic-co-glycolic acid)/poly(vinyl alcohol) composite as a wound dressing [65], and El-Aassar et al. prepared an Ag NP-poly(glycolic acid)/hyaluronic acid nanofiber wound dressing [66]. Those wound dressings all have good performance in promoting wound healing. For many Ag NPs loaded polymer matrices, hydrophilic materials are preferred, in view of the requirement for them to release silver ions and be in contact with the target bacteria. The combinations of essential oils and Ag NPs have been incorporated into electrospun fiber membranes and explored in wound dressing applications. Sofi et al. electrospun a mixed solution composed of Ag NPs, LO and polyurethane and prepared a fiber mat used for wound dressing [59]. Phan et al. loaded orange essential oil and Ag NPs on a treated electrospun CA fiber membrane through the soaking method [67]. In antibacterial tests, they all demonstrated good antibacterial performance.

**Figure 1:**
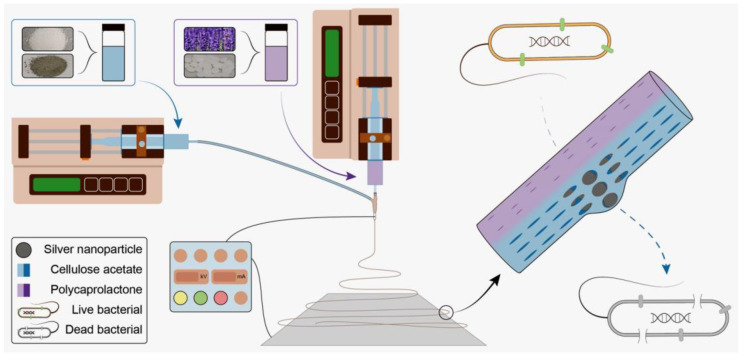
Schematic diagram of preparing solutions, side-by-side electrospinning process and Janus fiber.

The explorations in uniaxial electrospinning have been reported many times; however, Janus fiber from side-by-side electrospinning is short on research. In this paper, an organic antibacterial material, LO, and inorganic antibacterial material, Ag NPs, were combined into a dressing potential material. A ‘one-step’ method was implemented to prepare PCL-LO//CA-Ag NPs Janus fibers (Figure 1). The hydrophobic polymer PCL and swelling polymer CA were used as excipients. Lavender oil and Ag NPs were loaded into PCL and CA, respectively. Janus fibers were successfully fabricated with good performance both in terms of their antibacterial and water-resistant properties. The fiber mats, thus, have significant potential in wound dressing applications, and Janus electrospinning is demonstrated to be an efficient platform for preparing functional fiber materials.

## 2. Materials and Methods

### 2.1. Materials

Cellulose acetate (CA, average M_n_~30,000, 39.8 wt.% acetyl, 1.3 g/mL) and polycaprolactone (PCL, average M_n_~80,000, 1.145 g/mL) were purchased from Sigma Aldrich, St. Louis, Missouri, USA. Lavender oil (LO, 0.882 g/mL) was obtained from Shanghai Macklin Biochemical Co., Ltd., Shanghai, China. Silver nanoparticles (Ag NPs, size: 60~150 nm) were provided by Jiangsu Xianfeng Nanomaterials Technology Co., Ltd., Xianfeng, China. Solvents, 1,1,1,3,3,3-hexafluoro-2-propanol (HFIP) and trichloromethane (TCM), were obtained from Shanghai Macklin Biochemical Co. Ltd., Shanghai, China, and are analytically pure.

### 2.2. Preparing Janus Fibers

**Figure 2:**
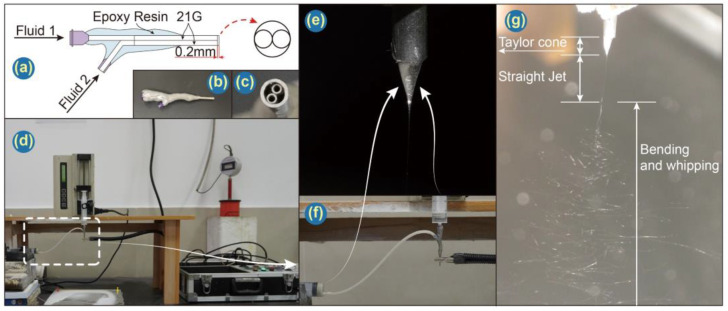
(**a**) Schematic and (**b**,**c**) photographs of the Janus; (**d**) image of the complete electrospinning apparatus; (**e**) Photograph of the Taylor cone obtained with two working fluids; (**f**) photograph of the working fluids; (**g**) digital image of the electrospinning process.

Janus electrospinning processes were conducted with a home-made spinneret as depicted in Figure 2a. Four different spinning solutions were prepared (Table 1). All solutions were prepared in a similar fashion. To take F4 as an example, 0.6 mL LO was added into a pre-prepared 10% (*w*/*v*) PCL solution (1.0 g PCL in 10 mL TCM) and subjected to magnetic stirring overnight. 1.0 g CA and 0.4 g Ag NPs were dispersed into 10 mL HFIP under ultrasonication for 30 min before use.

Janus electrospinning has been detailed in our previous work [68]. Briefly, two working fluids were loaded into syringes and dispensed by separate pumps (KDS100, KDS200, Cole Parmer, Chicago, IL, USA) into a home-made spinneret. A high voltage electrostatic field was generated by a high-voltage electrostatic generator (ZGF-60 kV/2 mA, Huatian Electric Power Automation Co. Let., Wuhan, China), and the product was collected on aluminum foil. The collection distance and voltage were set to 20 cm and 7.5 kV, respectively. Both flow rates were set at 1.0 mL/h. Images of the apparatus are presented in Figure 2b–g.

### 2.3. Characterization

#### 2.3.1. Morphology and Structures

A Quanta FEG450 field emission scanning electron microscope (SEM, FEI Corporation, Hillsboro, OR, USA) was used to examine the morphology of the fibers. Before they were detected, samples were cut from collectors, pasted on the sample table without removing aluminum foils to ensure fibrous membranes were flat, and coated with gold for 120 s to make them conductive. The average diameter was calculated using ImageJ (National Institutes of Health, Bethesda, MD, USA) to measure 100 randomly selected locations in the images. The internal structure of the fibers was determined using a JEM 2200F transmission electron microscope (TEM; JEOL, Tokyo, Japan).

#### 2.3.2. Physical and Chemical States

An AXS X-ray powder diffractometer (XRD; Bruker, Bremen, Germany) was used to collect XRD patterns within the 2θ range of 10~80°. A Spectrum 100 spectrometer (Perkin-Elmer, Waltham, MA, USA) was used to collect attenuated total reflectance-Fourier transform infrared (ATR-FTIR) data from 500 cm^−1^ to 4000 cm^−1^ with a resolution of 4 cm^−1^.

#### 2.3.3. Thermogravimetric Analysis

Thermogravimetric analysis (TGA) was used to explore the degradation temperature of the membranes. A sample weight between 6 and 8 mg was placed into a 6.5 × 4 mm alumina crucible. These tests were carried out under a nitrogen atmosphere while samples were heated at a rate of 5 °C per minute from 50 °C to 450 °C.

#### 2.3.4. Wetting and Moisture Retention Studies of Nanofiber Mats

Wetting studies were carried out using interfacial tension measuring apparatus (JC2000C1, Shanghai Zhongchen Digital Technology Apparatus Co., Ltd., Shanghai, China). A camera was employed to monitor changes in a drop of water (~0.5 μL) placed on each fiber mat. Built-in software was used to measure the water contact angle (WCA). Each sample was repeated six times, and the results were recorded as mean ± S.D.

In moisture retention tests, 0.5 g of fiber membrane was immersed into 200 mL deionized water for 1 h, taken out of the membrane with overloading water and drained for 2~3 min under natural conditions. Then, the centrifuge tube was put containing moist membrane into a centrifuge (LC-LX-L40B, Shanghai Lichen Instrument Technology Co., Ltd., Shanghai, China) with 1000× *g* centrifugal force for 15 min. Then, the water was poured out, and then the centrifuge tube was put back for another 15 min centrifugation. Then, the membrane was put into a pre-weighted reagent bottle, whose mass is *A*. After that, the membrane with the reagent bottle opened was placed on a heater set at 37 °C, and they were weighed at a predetermined time. Their mass was recorded as *B_n_* each time except for the mass at last time, which was marked as *B_end_*. Moisturizing efficiency (*M*) was calculated through the following equation. This test was repeated six times, and the results were recorded as mean ± S.D.

(1) $$M=\frac{B_{n}-B_{end}}{B_{end}-A}$$

#### 2.3.5. Antibacterial Activity

The antibacterial activity of the PCL-LO//CA-Ag NPs Janus fibers was determined against Gram-positive S. aureus and Gram-negative *E. coli* using a disk diffusion assay. Three 6-mm-diameter circular samples of each fiber formulation were placed on Luria-Bertani agar plates which had been uniformly coated with S. aureus or E. coli. The plates were incubated for 24 h at 25 °C, photographed, and the zones of inhibition measured. Results are reported as mean ± SD (*n* = 3).

## 3. Results and Discussion

### 3.1. Morphology and Structure of Janus Fibers

**Figure 3:**
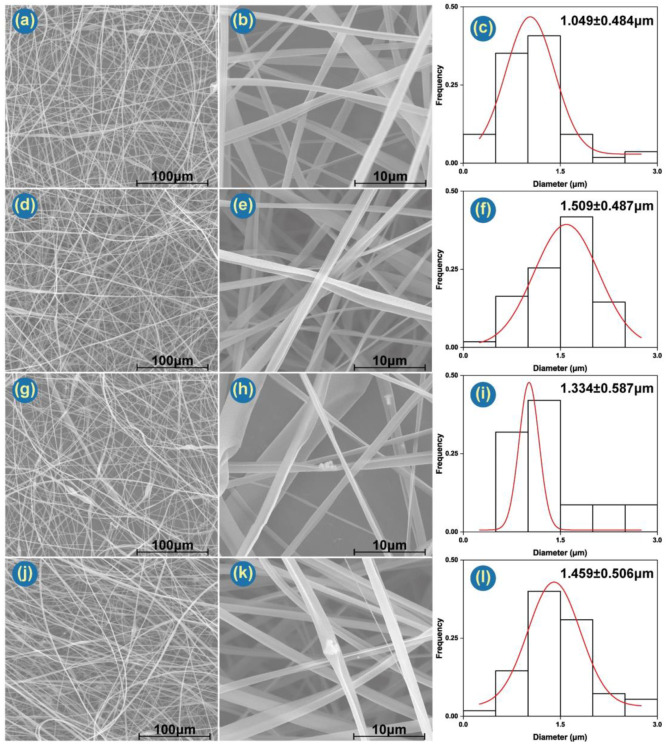
SEM images of the fibers: (**a**,**b**) F1; (**d**,**e**) F2; (**g**,**h**) F3; (**j**,**k**) F4. (**c**,**f**,**i**,**l**) are the distributions of fiber diameter determined from (**b**,**e**,**h**,**k**), respectively.

The Janus electrospinning processes utilize a macroscopic spinneret to fabricate microscopic fibers with a side-by-side structure. The diameters of the fibers are: F1 1.049 ± 0.484 μm, F2 1.509 ± 0.487 μm, F3 1.334 ± 0.587 μm and F4 1.459 ± 0.506 μm. F1 possessed the smallest average diameter and standard deviation. F3, the fiber containing Ag NPs in CA on one side and blank PCL on the other, has markedly less regular morphology than the others. Ag NPs can be clearly observed in the F3 and F4 fibers (Figure 3h,k). These images also indicate that the Ag NPs tend to aggregate, but this should not be detrimental to an antibacterial wound dressing.

**Figure 4:**
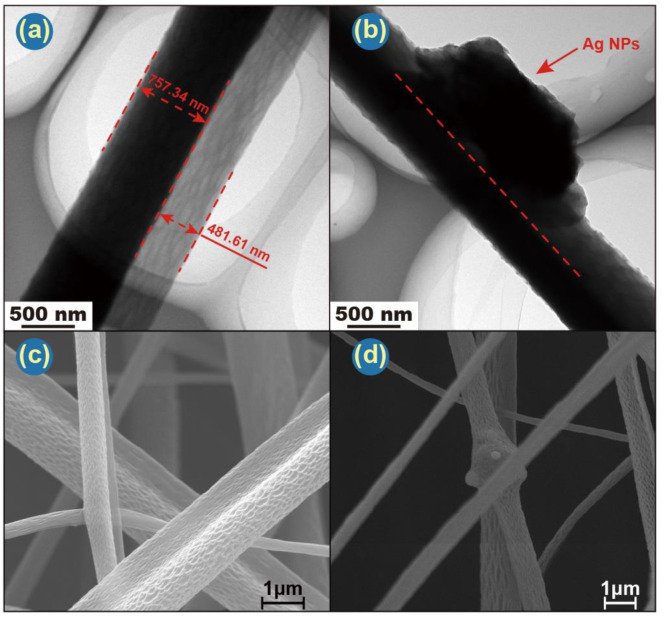
TEM images of (**a**) F1 and (**b**) F4. Scale bars are 500 nm. Enlarged SEM images of (**c**) F2 and (**d**) F3. Scale bars are 1 μm.

Combined with SEM and TEM images, Janus’s structure can be clearly observed. TEM images (Figure 4a,b) also depict representative Janus structures for both F1 and F4. F1 has a PCL side of ca. 757 nm and a CA side of 482 nm. Ag NPs in F4 are clearly visible in Figure 4b. Careful observation of the SEM (Figure 3b,e,h,k and Figure 4c,d) and TEM images (Figure 4a,b) reveals that there are many pores on the surface of both sides of the fibers. Figure 4c indicates the pore on the CA side are bigger and deeper than those on the PCL side. This may be governed by two parameters: solvent evaporation and polymer diffusion before fiber collection. According to Ref. [69], moderate and poor solvents for a given polymer cause nonuniformity in the polymer solution, hampering diffusion during the drawing process and resulting in a rough morphology.

The Ag NPs are clearly distributed throughout the CA side of the fibers (Figure 4d). The antibacterial ability of Ag NPs comes both from the NPs themselves and also from silver ions leached from them [70]. Both can induce oxidative stress, which will produce reactive oxygen species (ROS) and free radicals [71]. The presence of Ag NPs, which are exposed to the same humid environment as bacteria, should result in both the Ag NPs and freed Ag^+^ ions being able to interact with target bacteria, thus resulting in a potent antibacterial performance.

### 3.2. Formation Process of Janus Microfiber

**Figure 5:**
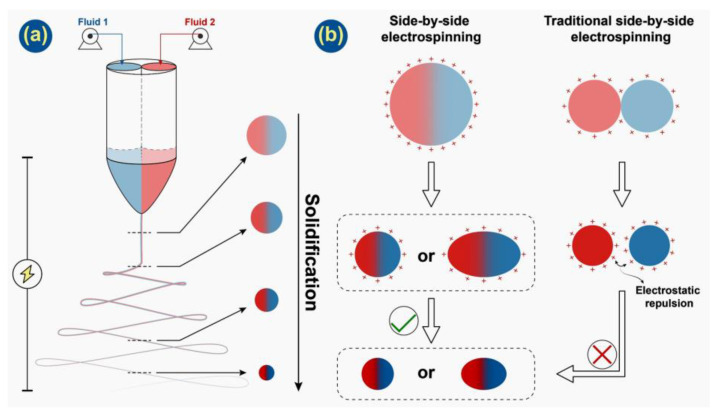
(**a**) Solidification process of working fluid along with side-by-side electrospinning; (**b**) charge distribution on the surface of this work compared with traditional side-by-side electrospinning.

The strategy in preparing Janus microfiber through a home-made spinneret with a protruding part that can promote the contact of two working solutions is traceable [41]. In the work reported by Fei et al., two parallel stainless needles were nested into a pipette, which is used to prolong the contact course between two working solutions. Through a long journal of two working solutions, a Janus Taylor cone can easily be formed. However, this spinneret may not be suitable for dilute solutions since they will blend into a mixture during this journal with low flow rates. In this work, a short protrude part was applied for the balance among mixing problems and contact purpose of working solutions. After working solutions flow through their capillary needle separately, there is a short period for the two working solutions to contact each other before forming the Taylor cone. Along with the three stages of the electrospinning process, Taylor cone, straight jet and bending and whipping, a working fluid composed of two solutions was solidified under a high voltage electrostatic field (shown in Figure 5a).

In Figure 5b, illustrations of the charge distribution of two different side-by-side electrospinning processes are provided. In traditional side-by-side electrospinning, the Taylor cone is formed by two columns of fluids with a small contact area. In the following stages, they would be separated by electrostatic repulsion most likely and would not form Janus fiber effectively. However, in this work, a home-made spinneret will provide effective contact between working solutions before the Taylor cone is formed. Even though Janus microfiber has been successfully prepared by using this spinneret, in this work, a cross-section of microfibers obtained tends to deform into oval from round. That can be easily observed in SEM images. There are some reasons. As PCL and CA were dissolved into TCM and HFIP, respectively, they have different conductivity, which will result in asymmetrical charge distribution of two sides. The diverse volatilization rates of two solvents also affect Janus microfibers’ formation. With their combined effect, the working fluid can be stretched into an oval shape by electrostatic repulsion. Besides F1, other Janus fibers will experience a similar course because essential oil and inorganic nanoparticles have little influence on the electrospinning process except for the morphology of fibers. Additionally, the single-fluid blending electrospinning is facile to encapsulate a certain drug with a high encapsulation efficiency [72,73], which often has few influences on the preparation of nanofibers. Similarly, the side-by-side electrospinning process is able to encapsulate two different types of active ingredients within each side of the resultant Janus structures effectively.

### 3.3. Physical Form and Compatibility

Different theoretical amounts of Ag NPs and LO in four Janus fibers have been listed in Table 2. However, with the volatilization of LO during the electrospinning process, there would be a lower weight percent in F2 and F4 than theoretical values.

**Figure 6:**
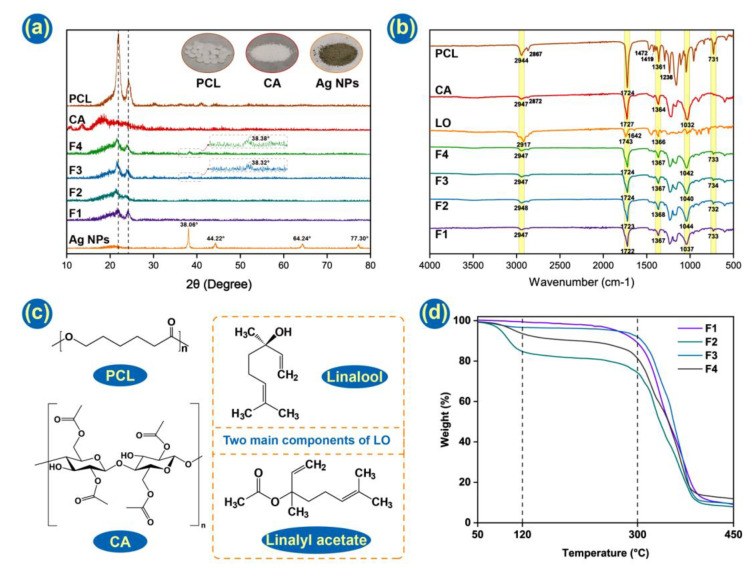
(**a**) XRD patterns of the raw materials and fibers. Inset: photographs of the raw materials; (**b**) FTIR results of raw materials and nanofibers; (**c**) Structural formula of PCL, CA and two main components of LO; (**d**) TGA diagram of four prepared fiber membranes.

The physical form of the raw materials and fiber mats was characterized by XRD. From Figure 6a, it can be seen that PCL possesses two strong Bragg reflections in the region of 20°~25°, consistent with its semicrystalline structure. The intensities of these were weakened in four nanofiber mats, which might be indicative of reduced crystallinity. This is logical since electrospinning can solidify a solution very rapidly, which means there is minimal time available for the organization of molecules to form a crystalline structure. CA demonstrates a broad halo in its XRD pattern, typical of an amorphous material. The Ag NPs indicate the expected Bragg reflections at 38.06° (111), 44.22° (200), 64.24° (220), and 77.30° (311). These are consistent with the literature structure PDF#04-0783. F3 and F4 indicate faint Ag NP reflections at 38.38° and 38.82°, confirming successful loading into the formulation.

The FTIR data is provided in Figure 6b and chemical structures in Figure 6c. In this test, the compatibility of components and interactions among them in Janus fiber have been explored. PCL demonstrates absorbance peaks at 1361, 1419 and 1472 cm^−1^ arising from bending vibrations of -CH2, at 2867 and 2944 cm-1 (symmetric and asymmetric -CH2 stretching), and at 1236 cm^−1^ (-COO vibration). CA has peaks at 2947 and 2872 cm^−1^ (C-H stretching) and 1032 cm^−1^ (C-O-C bending). These bands, in addition to the very distinct stretching of the ester C=O groups in CA and PCL from 1724–1727 cm^−1^, can all be seen in the spectra of the fibers [74]. The Ag NPs do not show appreciable absorbance in the FTIR and, thus, cannot be detected here. Absorbance bands at 2917 cm^−1^ (C-H stretching) and 1642 cm^−1^ (C=C stretching) can be seen in LO but not in the fibers’ spectra. However, other bands distinctive of LO, such as linalyl acetate, camphor and lavandulyl acetate carbonyl groups, can be seen confirming successful loading [59].

### 3.4. Thermogravimetry

It is clear that LO is electrospun into microfibers, and the perfume of LO is spread from membranes that can be smelt. However, as LO is uniformly dispersed into the PCL side in F2 and F4, there are few characteristic peaks of LO that can be distinguished in FTIR curves. Thus, TGA experiments were conducted, finding that LO-loaded fibrous membranes, F2 and F4, performed a distinctive weight loss from 70 °C to 120 °C. It is consistent with previous research that some essential oils can be fully removed at around 130 °C [75,76]. That indicates LO has been successfully incorporated into Janus fibers. Even though the value of weight loss does not consist of a theoretical value listed in Table 2, the content of LO should be all essential oil carried by fiber membranes because of the glass transition temperature of PCL at around 70 °C. When the temperature came to 70 °C, PCL would transform into a new physical state, which could not hold LO well. It is the volatilization during the electrospinning process that causes LO loss.

Before the temperature reached 300 °C, F1 and F3 had a small loss of weight, which was attributed to moist removal. All Janus fibers displayed a big weight loss from 300 °C to 400 °C. That is caused by the degradation of the polymer matrix as the degradation temperatures of PCL and CA are 364 °C and 335 °C, respectively [77,78,79]. In the end, the temperature rose to 450 °C, and all-fiber membranes lost around their 90% weight and came to a new plateau. The addition of Ag NPs performed little influence on TGA curves [66].

### 3.5. Wetting Ability and Moisture Retention Tests

Hydrophobicity is an important function for a wound dressing, preventing ingress of water from outside, which may cause infection and inflammation. Thus, water contact angle tests were performed to reveal the hydrophilic/hydrophobic properties of the fibers. The WCA of all the fibers exceeded 90°, showing they are all hydrophobic. The blank fiber (F1) has a WCA of 110.1 ± 3.7°, while F2~F4 have greater contact angles of 121.2 ± 7.1°, 119.3 ± 4.3°, 120.8 ± 4.3°, respectively. All the fibers are, thus, very hydrophobic and suitable for wound dressing applications, which will effectively protect the wound from infection. Although the fibers are initially hydrophobic, the presence of CA should permit them to absorb water and thus take up the exudate from a wound.

**Figure 7:**
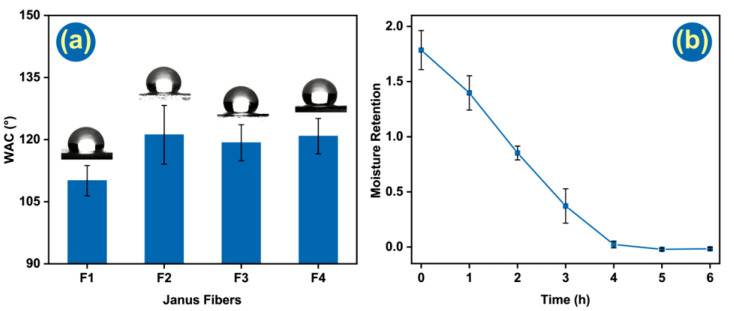
(**a**) Water contact angle tests of four kinds of Janus fiber; (**b**) moisture retention test for F1.

To eliminate the deviation brought by LO and Ag NPs release from Janus fiber, F1 was the only one tested in moisture retention. As polymers are the major part of absorbing and storing water, they can represent their water-saving property. After 30 min centrifugal process at 1000× *g*, superfluous water was removed. In the beginning, Janus fiber membranes can hold 1.786 ± 0.177 times its weight in water. Along with heat preservation time increasing, the moisture retention rate was reduced gradually. In Figure 7b, it is clear that water retained in the fibrous mat was gradually removed in four hours. That property can maintain a moist environment, which is a benefit for the wound healing process.

### 3.6. Antibacterial Tests

**Figure 8:**
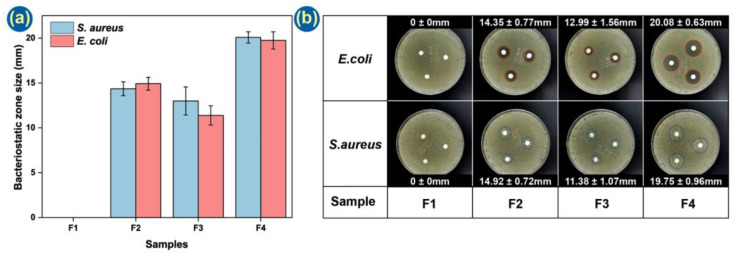
Antibacterial effects of the formulations. (**a**) Histogram and (**b**) photographs of the zones of inhibition.

Another important property of a wound dressing is antibacterial performance. Eliminating bacteria in the wound in a timely fashion can effectively avoid infection and inflammation. The presence of Ag NPs in the CA side of the fibers should provide a long-term protective effect. Figure 8 demonstrates that the F1 membrane has no observable antibacterial performance, but the other formulations demonstrate clear zones of inhibition and thus are able to prevent bacterial growth. The effects are similar against both gram-negative (*E. coli*) and gram-positive (*S. aureus*) bacteria. Formulation F4, carrying Ag NPs on the CA side and LO on the PCL side, demonstrates the greatest antibacterial properties.

**Figure 9:**
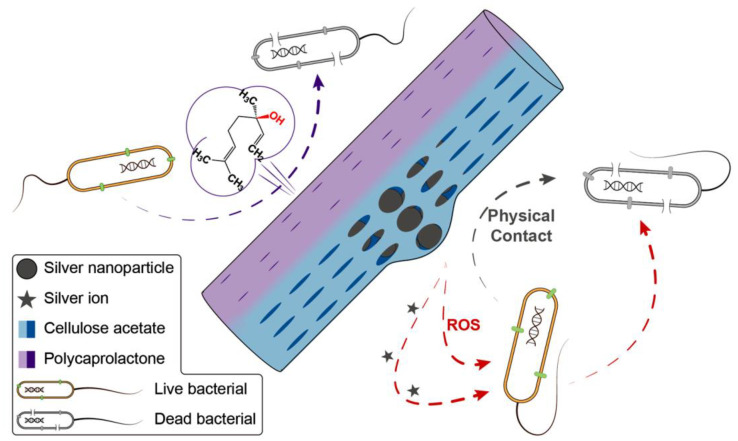
Antibacterial mechanism of prepared Janus nanofiber.

The mechanisms underpinning the antibacterial performance of the Janus fibers are illustrated in Figure 9. Linalool and linalyl acetate are the two main components of LO [80,81], with linalool having verified antibacterial properties. As the O atom of the hydroxy group within linalool has strong electrophilicity and free radical affinity, it can disrupt the intramolecular hydrogen bonding arrangement in protein during the antibacterial process [82]. The Ag NPs can release silver ions and generate ROS. These can destroy the bacterial cell membrane and increase its permeability [70]. Subsequently, silver ions can enter the bacteria, suppress respiration and cause leakage of the cellular contents [63]. Both processes can lead to bacterial death, and hence, the combination of LO and Ag NPs provides the most effective antibacterial performance.

## 4. Conclusions

In this research, Janus fibers loaded with LO and Ag NPs were prepared through a side-by-side electrospinning process. A home-made spinneret was used for enlarging the contact area of the working fluids prior to ejection from the spinneret. All the fibers generated have diameters around 1.0~1.5 μm. Ag NPs and LO were successfully loaded into the fibers, with the former in a CA-based compartment and the latter in a PCL compartment. There are many pores on the CA surface, allowing access to the Ag NPs by aqueous media. Wetting and antibacterial tests demonstrate the LO and Ag NPs loaded Janus fiber has good hydrophobic performance (with a WCA of 120.8 ± 4.3°) and strong antibacterial properties (20.08 ± 0.63 mm inhibition zone for E. coli and 19.75 ± 0.93 mm for S. aureus). The antibacterial properties of this material arise from the synergistic combination of LO and the Ag NPs, which can cause bacterial death through a number of mechanisms. The electrospinning process developed in this work provides an effective platform for preparing functional Janus fiber membranes.

## Figures and Tables

**Table 1:** Details of the working solutions used in this work.

NO.	Fluid1	Fluid2
F1	10% (*w*/*v*) PCL	10% (*w*/*v*) CA
F2	10% (*w*/*v*) PCL + 6% (*v*/*v*) LO	10% (*w*/*v*) CA
F3	10% (*w*/*v*) PCL	10% (*w*/*v*) CA + 4% (*w*/*v*) Ag NPs
F4	10% (*w*/*v*) PCL + 6% (*v*/*v*) LO	10% (*w*/*v*) CA + 4% (*w*/*v*) Ag NPs

**Table 2:** Theoretical amounts of Ag NPs and LO in four different nanofibers.

NO.	Fluid1	Fluid2	LO (*w*/*w*, %)	Ag NPs (*w*/*w*, %)
F1	10% (*w*/*v*) PCL	10% (*w*/*v*) CA	0	0
F2	10% (*w*/*v*) PCL + 6% (*v*/*v*) LO	10% (*w*/*v*) CA	20.9	0
F3	10% (*w*/*v*) PCL	10% (*w*/*v*) CA + 4% (*w*/*v*) Ag NPs	0	16.7
F4	10% (*w*/*v*) PCL + 6% (*v*/*v*) LO	10% (*w*/*v*) CA + 4% (*w*/*v*) Ag NPs	18.1	13.7

## Data Availability

Not applicable.
